# The Crosstalk between Tumor Cells and the Microenvironment in Hepatocellular Carcinoma: The Role of Exosomal microRNAs and Their Clinical Implications

**DOI:** 10.3390/cancers12040823

**Published:** 2020-03-29

**Authors:** Devis Pascut, Muhammad Yogi Pratama, Niem V.T. Vo, Rina Masadah, Claudio Tiribelli

**Affiliations:** 1 Fondazione Italiana Fegato—ONLUS, Liver Research Center, Basovizza, 34149 Trieste, Italy; yogi.pratama@fegato.it (M.Y.P.); ctliver@fegato.it (C.T.); 2 Center for Molecular Biomedicine, University of Medicine and Pharmacy at Ho Chi Minh City, Ho Chi Minh City 70000, Vietnam; thanhniem2611@ump.edu.vn; 3 Department of Pathology Anatomy, Hasanuddin University, Makassar 90245, Indonesia; rinamasadah@med.unhas.ac.id

**Keywords:** exosomes, miRNA, exosomal miRNA, hepatocellular carcinoma, liver cancer, HCC, tumor microenvironment, TME

## Abstract

The communication between hepatocellular carcinoma (HCC) cells and their microenvironment is an essential mechanism supporting or preventing tumor development and progression. Recent evidence has identified extracellular vesicles (EVs) as one of the mechanisms mediating paracrine signaling between cells. Exosomes, the most described class of EVs, deliver proteins, mRNAs, noncoding RNAs, DNA, and lipids to recipient cells, also at remote distances. MicroRNAs (miRNAs), as part of the non-coding RNA exosomal cargo, have an important role in regulating cellular pathways in targeted cells, regulating several processes related to tumor progression invasion and metastasis, such as angiogenesis, immune-escape, epithelial-to-mesenchymal transition, invasion, and multi-drug resistance. Accumulating evidence suggests exosomal miRNAs as relevant players in the dynamic crosstalk among cancerous, immune, and stromal cells in establishing the tumorigenic microenvironment. In addition, they sustain the metastasic niche formation at distant sites. In this review, we summarized the recent findings on the role of the exosome-derived miRNAs in the cross-communication between tumor cells and different hepatic resident cells, with a focus on the molecular mechanisms responsible for the cell re-programming. In addition, we describe the clinical implication derived from the exosomal miRNA-driven immunomodulation to the current immunotherapy strategies and the molecular aspects influencing the resistance to therapeutic agents.

## 1. Introduction

In solid tumors, cancer cells are surrounded by an extracellular matrix (ECM) that supports the tumor vasculature and a wide-range of cells, including cancer-associated fibroblasts (CAFs), lymphocytes, myeloid cells, and others, which coexist in a dynamic and adaptive environment [1]. Interactions among different cell populations often have a tumor-promoting role, fostering cancer cell growth from the initial phases of carcinogenesis. This supportive environment is maintained by a complex network of intercellular communications driven by cytokines, chemokines, growth factors, and extracellular vesicles (EVs). EVs are now emerging as one of the key players in the cell-to-cell mechanisms since they are able to deliver multiple and complex information to recipient cells [2]. Among the different types of EVs produced by cancer cells, such as microvesicles and large oncosomes, exosomes represent a widely studied class; they are disc-shaped vesicles ranging from 30–150 nm in size, and contain a molecular cargo consisting of proteins, lipids, and nucleic acids [3,4]. One particular class of non-coding RNAs in the exosomal cargo is represented by microRNAs (miRNAs), short non-coding RNAs (19–22 nt) usually involved in the regulation of the cellular pathway in both physiological and pathological conditions, including cancer [5]. Recent evidence highlights the capacity of exosomal miRNAs to regulate molecular pathways in recipient cells, thus affecting many aspects of tumorigenesis and tumor progression, such as angiogenesis, immune-escape, epithelial-to-mesenchymal transition (EMT), invasion, and multi-drug resistance. These aspects have also been also explored in hepatocellular carcinoma (HCC), which is now one of the deadliest cancers worldwide [6]. Because of the heterogeneity of the disease, both in terms of molecular alterations and cell populations constituting the so-called tumor microenvironment (TME), it has become of great importance the understanding of the complex exosome-mediated cell-to-cell communication networks in order to elucidate the mechanism fostering tumor cell growth. In this review, we summarize the recent findings on the role of the exosome-derived miRNAs in the cross-communication between tumor cells and different hepatic resident cells, focusing on the molecular mechanisms responsible for the cell re-programming. In addition, we describe the clinical implication derived from the exosomal miRNA-driven immunomodulation to the current immunotherapy strategies and the molecular aspects influencing the resistance to therapeutic agents. 

### 1.1. The Tumor Microenvironment Consists of a Mass of Heterogeneous Cell Types

Cancers are not just masses of malignant cells but a complex “rogue” organs, in which numerous different cell types, such as fibroblasts, adipocytes, immune cells, and cells from tumor vasculature, participate in tumor homeostasis [7]. Tumor cells and non-transformed cellular elements establish a dynamic network system consisting of growth factors, cytokines, chemokines, EVs, and matrix remodeling enzymes playing a role in the intercellular communication [7]. The impaired function of stromal cells contributes to cancer cell sustainment, developing a favorable environment for tumor growth.

#### 1.1.1. Lymphoid Cells

Different T cell populations infiltrate tumor areas within the TME [7]. T cell populations have a fundamental role in tumor initiation, progression, and metastasis [8]. The different tumor-infiltrating lymphocyte (TIL) subtypes have an opposite role within the TME. Both CD8+ cytotoxic T cells (CD8+CD45RO+) and CD4+ T helper 1 (Th1) cells exert an antitumor activity, and thus their presence is associated to good prognosis [7,9]. On the contrary, CD4+ TH2, producing IL-4, IL-5, and IL-13, generally promote tumor growth and development [9]. The CD4+ regulatory T cells (Tregs) are characterized by the expression of Forkhead Box P3(FOXP3) and CD25+ and are commonly described as tumor-promoting TILs. They exert an immune suppressive function in TME by producing IL-10, transforming growth factor beta (TGF-β), and by cell-mediated contact through cytotoxic T lymphocyte antigen 4 (CTLA4) [7,10]. High levels of Tregs are associated with worse outcomes in many kinds of cancer [11,12], and numerous groups have reported that CD25+ T cell depletion significantly increased antitumor immunity in different mouse models [13]. Another particular class of TILs includes the γδ T cells, which show a potent cytotoxic activity against a wide array of malignant cells, including cancer stem cells; however, their role associated to the clinical outcome is still unclear [14]. 

B lymphocytes also have a role in controlling cancer growth. Activated B cells showed a significant IgG2b-dependent anti-tumor activity, however, a particular subset of B lymphocytes, known as regulatory B cells (Bregs), CD19+CD24hiCD38hi, are able to induce immune tolerance by producing high levels of IL-10 and/or TGF-β, inhibiting Th1 and promoting Treg activity [15,16]. 

IL-10 and TGF-β, together with IL-6 released in the TME, also inhibit natural killer (NK) and natural killer T (NKT) cells, therefore reducing their potent killing activity against tumor cells [17]. NK and NKT cells can induce granzyme/perforin-mediated apoptosis and Fas/Fasl-mediated cell death, and they produce different types of cytokines. The reduction of NK in HCC [18,19] facilitated the escape of tumor cells from immune surveillance, however, accumulating evidence revealed that the subset CD49a+ NK cells, a tissue-resident subpopulation of NK, are negative regulators of immune responses that are associated with a poor prognosis [20].

#### 1.1.2. Myeloid Cells

Tumor-associated macrophages (TAMs) are one of the major components of tumor stroma in HCC. Macrophages are multifunctional antigen-presenting cells categorized into two phenotypes: pro-inflammatory (M1) and anti-inflammatory (M2). M1 are activated by Interferon γ (IFNγ) and bacterial products, and they express high levels of IL-12, low levels of IL-10, and have an anticancer activity [21]. TAMs belong to the M2 subtype, and their presence is associated with poor clinical outcome [22,23]. In response to tumor cells or Th2-derived stimuli (IL-4, IL-10, IL-13, TGF-β) [24] macrophages differentiate to M2 TAMs characterized by the production of IL-4, IL-5, IL-10, C-C Motif Chemokine Ligand 2 (CCL2), CCL17, C-C Motif Chemokine Receptor 2 (CCR2), C-X-C Motif Chemokine Receptor 1 (CXCR1), CXCR2, and high levels of programming death-ligand 1 (PD-L1) to promote angiogenesis and immune system suppression [24]. M2 TAMs are responsible for extracellular matrix remodeling, angiogenesis, and immunosuppression, making them indicators of poor prognosis in several malignancies, including HCC [25]. 

Dendritic cells (DCs), as antigen processing and presenting cells, play a pivotal role in regulating the balance between CD8+ T cell-induced immunity *vs.* tumor tolerance. However, the hypoxic and inflammatory environment in the TME inhibits the capability of DCs to activate an adequate immune response to tumor antigens [21].

Contrasting evidence describes neutrophils as having pro-tumorigenic or antitumor function. In certain cases, they promote primary tumor growth and metastasis by releasing IL-8 [26]. Conversely, some evidence has highlighted the inhibitory role of these cells at the metastatic site where they exert a cytotoxic activity, which is able to partially counteract the cancer cell seeding into metastasic sites [27]. 

Other myeloid cells, also known as myeloid-derived suppressor cells (MDSCs), feature the ability to suppress CD8+ T cell antitumor immunity through the expression of nitric oxide synthase 2 (NOS2) and arginase 1 (ARG1) [28]. 

#### 1.1.3. Other Cells

The activated fibroblasts in the TME are named as cancer-associated fibroblasts (CAFs), and are the main source of collagen-producing cells, expressing α-smooth muscle actin (α-SMA), fibroblast activation protein (FAP), vimentin, and fibroblast-specific protein 1 (FSP-1). They represent the major stromal cell type with multiple roles in influencing tumor cell proliferation, migration, invasion, angiogenesis, immune escape, and drug resistance through an extended network of intercellular communication with tumor cells and other stromal cells [29].

Endothelial cells also play a fundamental role in sustaining tumor growth. Neo-angiogenesis is essential in providing oxygen and nutrients for tumor growth. This occurs through an intensive interplay between tumor cells and/or stromal cells and vascular cells, which involves several mediators, such as vascular endothelial growth factors (VEGFs), Fibroblast Growth Factor 4 (FGF4), and others [30]. Quiescent endothelial cells are activated by these mediators in the presence of hypoxia, and once the angiogenesis is turned on, cancer begins to grow and metastasize. 

Recent evidence has assigned a tumor-promoting role to adipocytes that assist the recruitment of malignant cells through the secretion of adipokines and induce the growth of malignant cells by providing fatty acids as fuel for the cancer cells [31].

### 1.2. Characteristics of Extracellular Vesicles 

EVs are produced and released by several cell types both in physiological and pathological conditions, and they can be found almost all biological fluids, such as blood, urine, bile, saliva, semen, cerebrospinal fluid, as well as ascitic fluid [32]. On the basis of their cellular biogenesis and characteristics, EVs are divided into three main groups: microvesicles (MV), apoptotic bodies, and exosomes [32]. 

However, a cancer cell-specific type of EVs, named large oncosomes, have been described [4,33]. They are much larger than the other types of EVs, having a diameter of 1–10 µ, containing several types of RNAs and proteins. Large oncosomes partially share the biogenesis pathway with MVs and originate from plasma membrane of cancer cells that have acquired an amoeboid phenotype [4].

MVs originate directly from the plasma membrane, having a heterogeneous size range around 50–1000 nm in diameter. The process that leads to MVs generation starts from the formation of outward buds in specific sites of the membrane, followed by fission and subsequent release of the vesicle into the extracellular space [34,35]. This process involves specific machinery in which ADP-ribosylation factor 6 (ARF6) plays a central role [34,36]. They have multiple biological functions depending on the cell type from which they originate and/or on the cargo content that includes proteins and RNAs, including miRNAs [37]. 

Apoptotic bodies derive from blebbing and membrane fragmentation during apoptosis. They have a variable dimension, usually larger than 500 nm. Their content is generally randomly packaged, however, there is some evidence proving some sorting of RNA and DNA into specific subpopulations of apoptotic bodies [38]. 

Due to their role in cell-to-cell communication, exosomes have in recent years witnessed a growing interest in many fields of research, including oncology. They are 30-150nm-sized vesicles originating from the intraluminal vesicles (ILVs) within the multivesicular bodies (MVBs) as part of the endocytic machinery known as late endosomes [3,39,40]. During this process, proteins, lipids, DNA, messenger RNAs, and non-coding RNAs (ncRNAs), including miRNAs, are selectively sorted and loaded into exosomes [41,42,43]. Exosome biogenesis, cargo sorting, and release is a complex mechanism reviewed extensively in Hessvik and Llorente [44]. Several proteins involved in exosome biogenesis, sorting, and release have been identified as exosome biomarkers, although they have a broad expression in other EVs. These markers include the proteins Tumor Susceptibility 101 (TSG101), ALG-2-interacting protein X (ALIX); syntenin; and the tetraspanins CD9, CD63, CD81, and CD82. Several studies profiled the RNA content of exosomes and identified thousands of different miRNA species, annotated into the ExoCarta database (http://www.exocarta.org/). In 2007, Valadi and colleagues [45] found exosomes able to transport and transfer mRNAs and miRNAs to recipient cells. Since then, convincing evidence has shown that cancer cells actively release miR-containing exosomes that are part of a complex regulatory communication network between cells. In 2011, Kogure and colleagues [46] firstly proved that the exosome-mediated miRNA transfer is an important mechanism of intercellular communication in HCC cells. Despite several studies performed in HCC, mechanisms and functions still have to be further elucidated to better clarify the role of exosomal miRNA transfer in this tumor. 

### 1.3. Exosomal miRNAs as Regulators of the Tumor Microenvironment 

**Figure 1:**
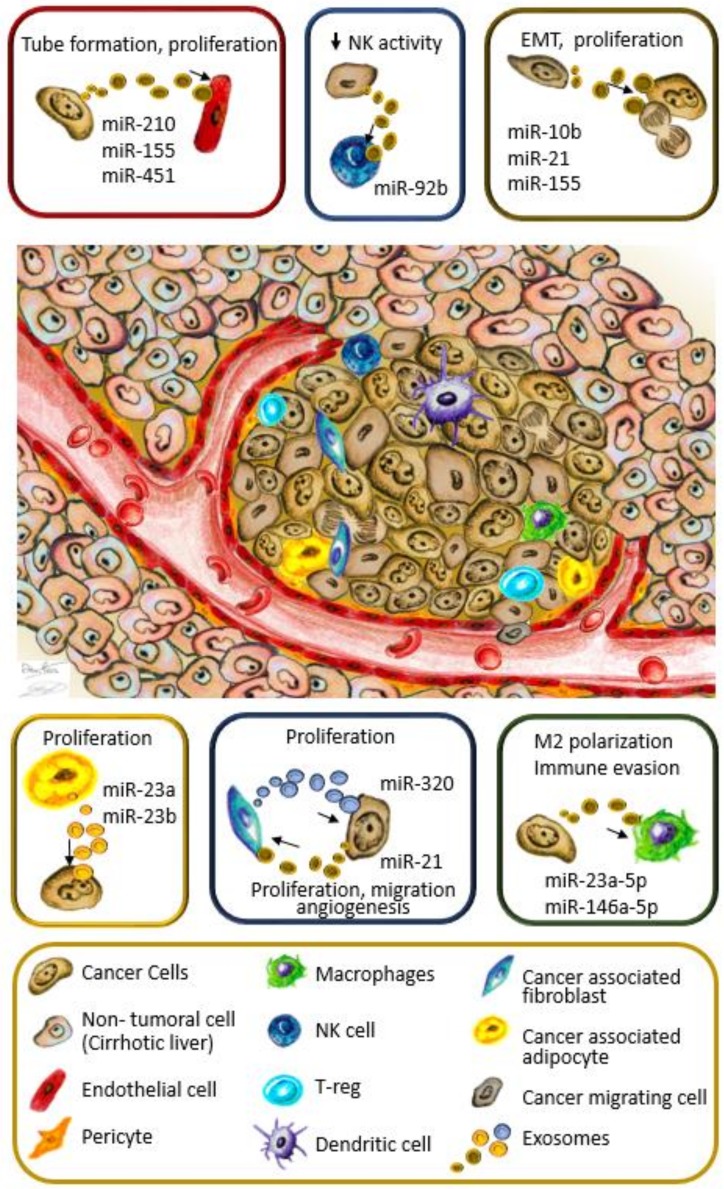
Intercellular communication in the tumor microenvironment. Different cell types reside within the tumor microenvironment (TME), infiltrating immune cells, both from innate and acquired immunity, cancer-associated fibroblasts (CAFs), cancer-associated adipocytes, and cells constituting tumor vessels. All these cells cooperate in a complex regulatory network fostering tumor establishment, growth, and metastasis, in which exosomal microRNAs (miRNAs) are emerging as relevant players in cell-to-cell communication. In colored boxes are the exosomal-miRNA exchanges between cells of the TME. The absence of miR-451 and miR-320 in exosomes fosters tumor growth, as described in the text. NK, Natural killer Cells; T-reg, Regulatory T cells.

Cancer nodules are a complex aggregation of many cell types constituting the TME. The dynamic interaction among the different cell population often have a tumor-promoting role, fostering cancer cell growth from the initial phases of carcinogenesis. This supportive environment is maintained by a complex network of intercellular communications driven by cytokines, chemokines, growth factors, and microvesicles. In particular, in exosomes released by cancer cells, hundreds of different miRNA species have been identified [46]. A selective mechanisms involving heterogeneous nuclear ribonucleoproteins (hnRNPs) [47,48] ensures the enrichment of specific miRNAs in the exosomal cargo [46] that, when internalized by recipient cells, contribute to the environmental modulation for HCC development, growth, and progression to metastasis (Figure 1). 

#### 1.3.1. The Transfer of HCC-Derived Exosomal miRNA Induces Neighbor Cancer Cells to Proliferate in the TME

The generally acidic TME of a solid tumor can be attributed to excessive glycolysis and poor perfusion [49,50]. Both primary and early malignant lesions consume high levels of glucose, even in the presence of oxygen, known as the “Warburg effect” [51]. In addition to cancer cells, stromal cells also contribute to the acidification of the environment [52] that has been proven to promote angiogenesis [53], EMT [54], invasion, and metastasis [55]. HCC cells grown in acidic conditions release exosomes containing high levels of miR-21 and miR-10b. The up-regulation of these two miRNA in cultured HCC cells is determined by the binding of Hypoxia-induced factor 1α (HIF-1α) and HIF-2α to the hypoxia response elements (HRE), located in the miRNA genes [56]. The internalization of exosomes enriched in miR-10b and miR-21 is able to increase proliferation in recipient HCC cells by increasing vimentin and Snail expression, while decreasing phosphatase and tensin homolog (PTEN) and E-cadherin [56]. In recipient cells, the increased vimentin expression and the synchronous E-cadherin repression are determined by miR-10b, targeting Kruppel Like Factor 4 (KLF4), which results in the activation of KLF11 and Small mother against pentaplegic proteins (Smads) to promote EMT [57]. At the same time, miR-21 suppresses PTEN, leading to the activation of AKT/ERK pathways and a consequent EMT [56]. Other experiments highlight the dual role of exosomal miR-21, released by cancer cells, in regulating PTEN expression in recipient Hep3B and HepG2 cells [58] (Table 1). In targeted cells, miR-21 downregulates Tet methylcytosine dioxygenases 1, 2, and 3 (TETs), responsible for the long non-coding RNA PTEN pseudogene 1 (PTENp1) demethylation [58]. The long non-coding RNA PTEN pseudogene 1 (PTENp1) acts as a miR-sponge, competing for the binding to miRNAs targeting PTEN, thus preventing PTEN downregulation [59]. The inhibition of TETs by miR-21 ensures stable PTENp1 methylation, which synergizes with the PTEN downregulation in promoting cancer cell growth both *in vitro* and *in vivo*. These findings explain the usually inversely related expression of miR-21 and PTEN in tumor specimens, in which miR-21 is up-regulated whereas PTEN is downregulated, as well as the higher miR-21 expression in serum of HCC patients [60,61].

In the cell-to-cell communication network, the role of other miRNA targeting PTEN is evidenced by the transfer of Hep-3B-derived exosomes, enriched in miR-155, to HepG2 cells. The downregulation of PTEN resulted in a Phosphatidylinositol 3 Kinase (PI3K)-AKT pathway activation, determining cell proliferation [62]. Although this paracrine regulatory network, highlighting the role of mir-10b, -21, and -155 in HCC, proliferation and EMT, have never been proven in the same experimental model, it may suggest a possible synergism of cancer cell-derived exosomal miRNAs in fostering cancer progression by enhancing neighbor cancer cell growth (Table 1, Figure 1). Limited experimental evidence exists about the role of exosomal mir-224 in HCC. The exchange of this oncomiR through exosomes, between cancer cells, can contribute to cancer proliferation by targeting the glycine *N*-methyltransferase (GNMT) [63], an oncosuppressor gene with multiple roles in preventing cancer [73]. 

#### 1.3.2. Cancer-Derived Exosomal miRNA Promote Angiogenesis by Targeting Endothelial Cells

Neo-angiogenesis is a critical step for tumor growth and metastasis because it supplies adequate nutrition and oxygen to the growing mass. HCC is a typical hypervascular tumor, in which angiogenesis plays a key role in sustaining cancer growth and metastasis, also contributing to the poor prognosis [74]. The proangiogenic signaling molecule vascular endothelial growth factor (VEGF) and its receptor VEGFR are the main drivers of neo-angiogenesis and are highly expressed in tumor tissues [75,76]. The binding of VEGF to its receptor on endothelial cell surface activates the AKTPI3K/MAPK pathway, resulting in proliferation, migration, and invasion of endothelial cells that lead to the formation and branching of new tumor blood vessels [77,78]. Although VEGF is the main soluble cytokine inducing angiogenesis, it is not the only regulatory factor. Recently, exosomal miRNA have been identified as other molecules influencing the vessel formation and growth in tumors. The highly expressed HCC cell-secreted exosomal miR-210 has a proven role in driving tubologenesis [67]. Exosomes containing miR-210, isolated from patients and from QGY-7703, Hep-G2, SK-Hep-1, and Huh7 cells, were able to promote tube formation of umbilical vein endothelial cells (HUVECs) by targeting SMAD4 and Signal Transducer and Activator of Transcription 6 (STAT6), negative regulators of angiogenesis (Table 1) [79]. In addition, QGY-7703-miR-210-derived exosomes induced a higher microvessel density (MVD) and larger tumors in xenograft models [67]. Interestingly, the levels of circulating miR-210 were correlated with bigger tumor size, advanced clinical stage, and higher MVD [67].

Hypoxia in the TME is a potent driver of tumor angiogenesis [80]. It induces VEGF expression, enhancing the formation of new blood vessels [81]. The hypoxic environment induces PLC/PRF/5 and Huh7 cells to secrete exosomes containing miR-155, which exerts a tube promoting action on HUVEC cells (Table 1, Figure 1). Although the direct target was not identified, the increased preoperative circulating exosomal miR155 was significantly correlated with early recurrence and poor prognosis [68]. Interestingly, experimental evidence proved the contribution of miR-155 in controlling HIF-1α expression under hypoxia to promote angiogenesis, although in other clinical settings [82].

Still uncertain is the role of exosomal mir-451a, previously named mir-451, in HCC. It is well established that miR-451a is downregulated in primary cancers and cancer cell lines [69]. The ectopic expression of miR-451a significantly reduced cell viability and migration of the SMMC-7721 cell line by targeting lipin 1(LPIN1). At the same time, cancer releases mir-451a-enriched exosomes that target endothelial cells, determining apoptosis and a reduced migratory potential [69]. Mir-451a, transferred into endothelial cells, can also exert its inhibitory effects by inhibiting the IL-6R-STAT3-VEGF signaling pathway that contributes in the reduction of angiogenesis (Table 1) [83]; however, this should be proven as a consequence of miR-451a exosomal transfer. In other experimental models, exosomal miR-451a can stimulate the T cell conversion into Th17 resulting in an accumulation of the immune cells in the TME that can promote angiogenesis, although in gastric cancer [84]. It has been hypothesized that miR-451 downregulation occurs during tumorigenesis; in this process, cancer cells eliminate miR-451 by exosomal release, which can, at the same time, contribute to sustaining a favorable TME by recruiting Th17 cells [84]. 

#### 1.3.3. CAFs Foster HCC Growth through Exosomal miRNA Exchange

CAFs are unique reprogrammed stromal cells with roles in cancer initiation, extracellular matrix remodeling, progression, pre-metastatic niche formation, and metastasis. CAFs secrete different tumor-supportive growth factors and nutrients to support cancer growth; in addition, they regulate the inflammatory environment during tumorigenesis [29,85]. In HCC, their origin remains controversial, as they can derive from activated hepatic stellate cells (HSC), from portal fibroblasts, or from transdifferentiation of hepatocytes through EMT, showing different phenotypes and biological function [29]. HCC cell-derived exosomes activate human stellate cell line LX2, improving their proliferation and migration. Their activation contributed to the growth of multiple subcutaneous tumors in nude mice [65]. The highly abundant miR-21 present in cancer cell-derived exosomes was proven to activate target HSC through PTEN inhibition and the consequent PDK1/AKT pathway activation (Table 1). In tumors generated by the injection of Huh7 cells and exosome-treated LX2 in nude mice, the cancer vascularization was increased compared to the control group treated with miR-21 inhibitor. In addition, CAFs were significantly enriched in tumor vessel proximity and showed an increased expression of VEGF-α, Matrix Metallopeptidase 2 (MMP2), MMP9, basic Fibroblast Growth Factor (bFGF), and TGF-β [65]. Finally, vascular endothelial cells cultured with exosome-treated CAFs increase their proliferation and formation of tubular structures [65]. These results described the existence of a positive feedback loop in which hepatic cancer cells are able to induce favorable conditions to their growth by establishing a HCC–CAF–endothelia cell axis. Zhang and colleagues (2017) [66] proposed a model in which HCC cells educated surrounding normal stromal cells to differentiate into CAFs by secretion of TGF-β and other factors. Once established, CAFs contributed to the tumor microenvironment by reducing the release of antitumor miR-320a in exosomes. In tumor-inhibiting conditions, exosomal miR-320a targets PBX Homeobox 3 (PBX3) in recipient cells, resulting in a reduction of phosphor-ERK1/2 and *N*-cadherin as well, as an increase of E-cadherin. The loss of miR-320a reverses these effects, resulting in tumor proliferation and EMT activation [66]. 

#### 1.3.4. The Interplay between Hepatocellular Carcinoma Cells and Adipocytes Creates a Favorable Microenvironment for Tumor Growth

A recent discovery involves the communication between liver cancer cells and adipocytes within the tumor microenvironment. Adipocytes have a significant role in providing support for cancer cell growth. In breast cancer, tumor cells induce the transformation of adipocytes into cancer-associated adipocyte (CAA) [86,87], characterized by a decreased content in intracellular lipids and late adipose markers, as well as in an increased expression of inflammatory cytokines and proteases. CAAs increase the secretion of free fatty acids (FAs) that are uptaken from cancer cells to sustain their growth [86]. In addition, CAA-derived exosomes transfer enzymes implicated in fatty acid oxidation (FAO) directly into cancer cells, further sustaining cell proliferation and transformation into a more aggressive phenotype [88]. These mechanisms might be of particular relevance in HCC, especially considering the increasing rate of Nonalcoholic Fatty Liver Disease (NAFLD)-derived HCC in recent years [89,90]. The crosstalk between adipocytes and HCC cells is still an emerging field of investigation. In a single study, miR-23a and miR-23b, encapsulated into exosomes derived from mature adipocytes, were able to promote the proliferation of BEL-7402 hepatocellular cancer cells (Table 1), upregulating the expression of HIF-1α, Glucose Transporter 1(GLUT-1) and VEGF *via* von Hippel–Lindau (VHL) inhibition [64]. The inactivation or loss of the VHL gene is generally associated with the development of tumors. It plays a role in HIF-1α ubiquitin-mediated degradation [91], and thus its downregulation, by miR-23a/b, ensures the proliferation of cancer cells. Other evidence showed that Hep-G2 exosomes activated several kinases-dependent signaling pathways (AKT, STAT5α, Glycogen Synthase Kinase-3 (GSK3) α and β, ERK1/2) and Nuclear Factor kappa-light-chain-enhancer of activated B cells (NF-κB))in adipocytes, promoting an inflammatory response, through IL-6, IL-8, and Chemoattractant Protein 1 (MCP-1). In response, adipocyte-derived exosomes promoted tumor growth *in vivo* [92]. Although the relationship with miRNAs was not investigated in this experimental model, it is reasonable to expect some implications of those Small Non-coding RNAs (sncRNAs) in this cross talk. 

#### 1.3.5. Exosomes Play a Critical Role in Modulating Cancer Immune Escape

Exosomes have been identified as important regulators of immune escape mechanisms in cancer [93]. They mediate the interaction between cancer cells and immune cells, as well as among different types of immune cells to induce immune evasion and tumor progression [93]. In HCC, endoplasmic reticulum (ER) stress plays an important role in tumor progression and in regulating immune cell function [94]. It has been reported that ER stress markers levels correlate with macrophage infiltration and PD-L1 expression in HCC [70]. ER stress causes HCC cells to release exosomes enriched in miR-23a-3p targeting M2 type TAMs. Once internalized by M2 cells, miR-23a-3p inhibit the expression of *PTEN*, resulting in the up-regulation of PD-L1 through PI3K-AKT pathway activation [95]. Similar results were obtained by transferring miR-146a-5p-enriched exosomes from HCC cancer cells (mice and human) into macrophages [71]. Human HepG2 cells or H7402-derived exosomes stimulated the differentiation of TPH-1 cells into the M2 subtype, characterized by the upregulation of CCL17, CCL2, and PD-L1 [71] (Table 1). These modulatory effects on TAMs lead cancer cells to create a favorable TME, inducing an immune-evasion mechanism by impairing CD8+ T cell function (Figure 1). 

NK cells are another major component of the immunological landscape within the human liver [96]. Liver-resident NK expresses CXCR6 and CD69 markers, which have crucial cytotoxic activity against cancer cells. In HCC, NK cells are diminished in tumors compared to non-tumor regions; in addition, they possess a defective IFN-γ and TNF-α secretion caused by the defective recognition of tumor cells, or by inhibitory effects from other immune or cancer cells [19,97]. In particular, cancer cells, such as Hep-3B, can release miR-92b-rich exosomes that exert a suppressive action on NK by deregulating CD69 [72], which is a cell surface costimulatory molecule determining cell proliferation, cytokine secretion, and cytotoxicity (Table 1) [98]. miR-92b is classified as an oncomir in HCC [99], showing high levels both in tissues and in serum samples. The recent discovery of the modulatory effect on NK cells attributes a dual role on this miRNA, fostering HCC progression. On one side it promotes proliferation by targeting SMAD7 in HCC cells [99], and on the other side, the released form ensures a favorable background for tumoral growth by inhibiting the tumor immune surveillance [72]. Cell-to-cell communication that involves immune cells assumes particular relevance in consideration of the new immunomodulatory therapies available for HCC. The understanding of the mechanisms involved in this complex regulatory network would be of special benefit for future improvements of immunotherapies in this cancer. 

### 1.4. The Cellular Crosstalk in the Tumor Microenvironment Promotes Metastasis

The metastasis cascade is a complex process involving dynamic interactions between cancer cells and TME. The first critical step, leading to the development of the invasive phenotype of the malignant cells, involves at least three processes: angiogenesis, which provides nutrients, oxygen, and the “route” for the migration; EMT, through which the tumor cells acquire a stem-like, aggressive, and invasive phenotype; and invasion, which enables cells to intravasate into the circulatory system to reach distant sites to establish the so-called pre-metastatic niche. The TME interacts dynamically and plays an important role in the promotion of metastasis by secreting cytokines and growth factors. The interaction between tumor cells and stromal cells is understood as one of the determinant factors in the occurrence of metastasis [100]. Several stromal cells such as CAFs, mesenchymal stem cells (MSC), TAMs, and others can be involved in this process by releasing cytokines and exosomes that trigger cell proliferation, EMT, and the remodeling of the extracellular matrix [29,100]. CAFs also induce an inflammatory process in TME and inhibit the activity of cytotoxic T lymphocytes [29,101]. In this scenario, exosomal miRNAs seem to play relevant roles as metastasis-promoting factors. In HCC, the high levels of serum miR-103a-3p are associated with patients with metastasic episodes [102]. This exosomal miRNA, released by liver cancer cells, is able to attenuate the junction integrity of recipient endothelial cells (HUVEC) by targeting adhesion molecules such as VE-cad, p120, and zonula occludens 1 (ZO-1), thus increasing the vascular permeability both *in vivo* and *in vitro* [102]. The subsequent tumor cell transendothelial motility process is promoted by exosomal miR-25-5p [103]. The horizontal transfer of miR-25-5p between cancer cells induces migratory characteristics in recipient cells by targeting leucine rich repeat-containing 7 (LRRC7) protein. LRRC7, also known as densin-180, is a transmembrane protein that binds and stabilizes cell motility proteins such as δ-catenin and *N*-cadherin, ZO-1, Ca2+/calmodulin-dependent protein kinase II, and α-actinin [103]. LCRR7 downregulation by exosomal miR-25-5p transfer results in cell migration and tumor self-seeding in different sites. 

The importance of CAFs as metastasis-promoting cells becomes evident with the experiments conducted by Fang and colleagues (2018) [104] providing the importance of cell-to-cell communication in fostering tumor cell migration and metastasis. The release of exosomal miR-1247-3p from cancer cells, known for their metastatic potential, was able to induce the expression of *IL-1β*, *IL-6*, and *IL-8* in recipient CAFs by targeting β-1,4-galactosyltransferases III (B4GALT3). B4GALT3 inhibits β1-integrin activation and stability by glycosylation. In this way, the downregulation of B4GALT3 by miR-1247-3p stabilizes β1-integrin that causes fibroblasts activation through the NF-κB pathway [104]. The conditioned media collected by CAFs was then able to enhance EMT, spheroid formation, and motility in cancer cells, both *in vitro* and *in vivo*. These data were supported by clinical evidence in which patients having episodes of lung metastasis showed higher levels of miR-1247-3p in serum exosomes compared to patients without lung metastasis [104].

### 1.5. Targeting Tumor Microenvironment Cells Using Exosomal miRNAs as a Future Antitumor Strategy 

Because of the importance of TME for tumor development, progression, and metastasis, the targeting of multiple cell types participating in tumor sustenance might represent a plausible antitumor strategy to be further investigated. The transferring of miR-142 and miR-223 expressed from macrophage-derived exosomes to HCC cells has been described as inhibiting the proliferation of cancer cells, thus opening a hypothesis as to whether harvesting and engineering exosomes from immune cells might be able to interfere with the progression of cancer cells [105]. In addition, the overexpression of miR-320a in CAFs has been reported as an effective method to produce miR-320a-enriched exosomes that can transfer this miRNA to HCC cells, thus suppressing proliferation, migration, and metastasis when binding to its direct target, PBX3 [66]. The same approach was used to down-regulate the expression of several key genes that induce proliferation and invasion of HCC cells by taking advantage of HSC-derived exosomes loaded with tumor suppressor miR-335-5p [106]. Another study provided a novel approach to pack miR-26a into exosomes to selectively target HCC cells through the scavenger receptor, resulting in the inhibition of cell migration and proliferation [107]. All these reports showed a feasible strategy to inhibit cancer growth by using naturally or modified exosomes loaded with specific sets of miRNAs; however, this is still an emerging approach that needs time to be improved. 

#### 1.5.1. Exosomal miRNAs in HCC and Their Implication for Current Therapies

The multitude of cell types in the TME not only cooperate to support cancer growth but also to enhance cancer cells resistance to therapies [108]. The multikinase inhibitor sorafenib was considered for many years as the first-line treatment for advanced HCC, yet showed unsatisfactory results [109]. Indeed, phenomena of resistance were reported in some cases, resulting in a reduction of therapy efficacy [110,111]. More recently, experimental evidence proves the role of miRNAs in the acquisition of sorafenib resistance [112]. Accumulating evidence showed that exosomal miRNAs participate in the sorafenib resistance mechanism. MiR-32-5p was found to participate in the acquisition of sorafenib resistance in cancer cells. Exosomes, released by resistant cancer, transferred miR-32-5p into sorafenib-sensitive HCC cells, activating the PI3K/AKT pathway, inducing multidrug resistance by modulating angiogenesis and EMT [113]. Because of the elevated levels of exosomal miR-32-5p in HCC patient serum, associated with poor prognosis [113], it can be hypothesized that resistant cancer cells can share their resistance with neighboring cells *via* miR-32-5p transfer, thus hampering sorafenib therapies. An opposite effect was described for exosomal miR-774 and miR-122 [114,115]. mir-774 was found to be decreased in exosomes derived from the patient serum or HCC cells resistant to sorafenib [114]. However, the restoration of miR-774 expression in cancer cells lead to a production of miR-774-enriched exosomes that, once transferred into resistant Hep-G2 cancer cells, rescued sorafenib sensitivity [114]. The crosstalk between adipose tissue-derived mesenchymal stem cells (AMSC) and cancer cells was able to sensitize cells to sorafenib, both *in vivo* and *in vitro* [115]. The injection of miR-122-enriched exosomes derived from AMSC into a mouse model improved the anti-tumor effect of sorafenib, thus underlining the potential use of exosomal miR-122 as an approach to reverse drug resistance [115]. Exosomal miRNAs involved in mechanisms of drug resistance might also find application as circulating biomarkers as predictive and prognostic factors [113,116]. 

#### 1.5.2. Exosomal miRNAs’ Future Implications for Immunotherapy in HCC

Immune checkpoint inhibitors such as antibodies against PD-L1 and PD-1 have been introduced into clinical practice for a number of cancers [117,118]. In HCC, double-blind randomized clinical trials studies have shown that immune checkpoint inhibitor therapy can provide favorable responses in patients with advanced HCC [119]. However, recent results from phase III CheckMate 459 and KEYNOTE-240 studies of PD-1-targeted antibodies for advanced HCC did not achieve statistical significance or superiority in the overall survival in comparison to sorafenib [120,121]. Thus, another prospective strategy is to combine immune-oncology agents with other immune-modulating elements in which exosomal miRNA might play a synergic role in immunotherapy; for example, the inhibition of miR-23a-3p or miR-126a-5p, or both, in cancer cells could prevent its exosomal release and thus the consequent macrophage PD-L1 expression [70]. This might represent a plausible strategy to down-regulate PD-L1 in tumors. 

Previous studies described that the excessive production of VEGF in response to the hypoxia state in TME may exert an immunosuppressive effect in tumors, promoting immunosuppression and enhance immune checkpoint molecule expression, thus decreasing the efficacy of PD-L1 and PD-1 inhibitor drugs [122,123,124]. Faivre et al. proposed that, using this rationale, targeting tumor hypoxia and VEGF might improve the efficacy of current immunotherapies [125]. Because of the role of several exosomal miRNA in promoting angiogenesis in the TME, especially in hypoxic conditions, it is plausible that the targeting of specific miRNAs, such as miR-210 and miR-155 [67,68], might interfere with the cellular crosstalk promoting angiogenesis. In this way, future molecular medicine practices can take advantage of exosomal cargo to deliver specific miRNA, counteracting the tumor promoting function of the TME.

### 1.6. Exosomal miRNAs as Potential Biomarkers in HCC 

EVs, including exosomes, released from cancer cells significantly differ from those released from non-cancerous cells, both in terms of quantity and content [126]. Because the selective loading of miRNA has multiple roles in the paracrine cell-to-cell communication, exosomal miRNAs are becoming a new source of potential biomarkers in HCC. Different studies have identified exosomal miRNAs with potential as diagnostic or prognostic biomarkers. One of the earliest studies that specifically isolated miRNAs from exosomes in HCC identified miR-21 as a candidate biomarker for the identification of patients with cancer among patients with chronic hepatitis B (CHB) or healthy subjects [127]. Interestingly, in this study, Wang and colleagues reported higher performances of exosomal miR-21 compared to its serum counterpart, underling the possible higher specificity for exosomal miRNAs as biomarkers. Following this study, many other investigations identified several miRNAs in exosomes as diagnostic (Table 2) or prognostic (Table 3) biomarkers in HCC.

## 2. Conclusions

Numerous studies have provided information regarding the significant role of exosomal miRNAs on regulating dynamic crosstalk between different cell populations in the TME (see Appendix A). It is evident that exosomal miRNAs transferred between cells will alter various molecular pathways that favor tumor progression, ranging from immune escape to multi-drug resistance towards HCC treatments. Therefore, exosomal miRNAs provide a new potential angle as a diagnostic and prognostic biomarker or as a target for a future antitumor strategy for HCC.

## Figures and Tables

**Table 1:** Exosomal miRNA involved in cell-to-cell communication in hepatocellular carcinoma (HCC).

Exo-Releasing Cells	miRNA	Recipient Cells	Target	Effect	Validated in Mouse Model	Reference
Hep-3B, SMMC-7721	miR-21, miR-10b	Hep-3B, SMMC-7721	n.d.	EMT proliferation	Yes	[56]
Hep-3B	miR-155	HepG2	PTEN	Proliferation	Yes	[62]
SNU-449	miR-21	HepG2, Hep-3B	TETs/PTEN/PTENp1	Proliferation	Yes	[58]
HepG2	miR-224	SKHEP1 (adenocarcinoma)	GNMT	Cell proliferation	No	[63]
Mature adipocytes	miR-23a, miR-23b	BEL-7402	VHL	Proliferation	Yes	[64]
LM3, 97H liver cancer cells	miR-21	LX2	PTEN/PDK1/AKT pathway	Proliferation, migration, angiogenesis	Yes	[65]
Primary CAFs	miR-320a downregulation	MHCC97-H	PBX3	Proliferation, metastasis		[66]
QGY-7703, Hep-G2, SK-Hep-1, Huh7, EXOs from HCC patients	miR-210	HUVEC	SMAD4, STAT6	Tube formation, high MVD and larger tumors	Yes	[67]
PLC/PRF/5, Huh7	miR-155	HUVEC	n.d.	Tube formation	No	[68]
SMMC-7721	miR-451	HUVEC	LPN1 (in cancer cells)	Increased cell death, decreased cell migration		[69]
Hep-G2, Hep-3B	miR-23a-3p	THP-1	PTEN	Immune evasion by PD-L1 overexpression	Yes, partially	[70]
Hep-G2, H7402	miR-146a-5p	THP-1	n.d.	M2 polarization, T cell dysfunction	Yes	[71]
Hep-3B	miR-92b	NK cells	CD69	Reduced NK activity	No	[72]

**Table 2:** miRNA as biomarkers for diagnosis of HCC.

miRNAs		Patients	Clinical Significance	Reference
Diagnosis				
miR-21	↑	30 HCC, 30 CHB, 30 healthy	Discrimination between HCC and CHB or LC	[127]
miR-30b-3p	↓	50 paired HCC tissues (non-tumor tissues)	Biomarker diagnosis and treatment HCC	[128]
miR-210-3p	↑	29 HCC	Biomarker for the risk of HBV-related HCC	[129]
miRNA-224	↑	9 HCC and 50 normal serum samples	Biomarker of diagnosis and prognosis of HCC patient	[63]
miR-718	↓	59 HCC	Predicting biomarker for recurrence after LT	[130]
miR-18a miR-221 miR-222 miR-224 miR-101 miR-106b miR-122 miR-195	↑ ↑ ↑ ↑ ↓ ↓ ↓ ↓	20 HCC vs. 20 CHB vs. 20 LC	Discrimination between HCC and CHB or LC	[131]
miR-10b-5p miR-18a-5p miR-215-5p miR-940	↑ ↑ ↑ ↑	28 healthy, 60 CLD, 90 HCC	miR-10b-5p biomarker for early stage HCC	[132]
miR-483-5p miR-133a	↑ ↑	20 HCC, 20 CHB, 20 healthy	Noninvasive diagnostic biomarkers for HCC	[133]
miRNA-26a miRNA-29c miRNA-21	↓ ↓ ↓	72 HCC, 72 LC, and 72 HBV	Diagnostic biomarkers for patients with HCC	[134]
miR-122↑ miR-148a↑ miR-1246↑		5 HCC vs. 5 LC	Diagnostic biomarker discriminating HCC from LC	[135]

CHB: chronic hepatitis B; HCC: hepatocellular carcinoma; LC: liver cirrhosis

**Table 3:** miRNA use as biomarkers for prognosis of HCC.

miRNAs		Patients	Clinical Significance	Reference
Prognosis				
miR-125b	↑	158 HCC vs. 30 CHB vs. 30 LC	Predicting biomarker for recurrence and survival	[136]
miR-638	↓	126 HCC	Poor prognosis marker for patients with HCC	[137]
miR-10b-5p miR-18a-5p miR-215-5p miR-940	↑ ↑ ↑ ↑	28 healthy, 60 CLD, 90 HCC	miR-215-5p: prognostic biomarker for HCC	[132]
miR-744	↓	68 HCC and 52 normal liver tissue samples	Proliferation and chemoresistance	[114]

CHB: chronic hepatitis B; CLD: chronic liver disease; HCC: hepatocellular carcinoma; LC: liver cirrhosis.

## Appendix A

A literature search was performed by four independent researchers in the PubMed database, using the keywords “hepatocellular carcinoma”, “exosomal microRNA”, “tumor microenvironment”, and “cellular crosstalk”. Only studies reporting clear evidence of miRNA transfer through exosomes were included in the literature research.
